# Dermal fibroblast cell‐derived exosomes for atopic dermatitis: In‐vitro test

**DOI:** 10.1111/srt.13382

**Published:** 2023-06-23

**Authors:** Kwangho Yoo, Nikita Thapa, Jongjin Lee, Youna Jang, Jung Ok Lee, Jaeyoung Kim

**Affiliations:** ^1^ Department of Dermatology Chung‐Ang University Gwangmyeong Hospital Chung‐Ang University College of Medicine Gyeonggi‐do Republic of Korea; ^2^ CK‐Exogene, Inc. New Drug Development Center Seoul Republic of Korea; ^3^ Department of Regenerative medicine Daesung Hospital Seoul Republic of Korea; ^4^ Department of Dermatology Chung‐Ang University, College of Medicine, Seoul Campus Seoul Republic of Korea

Dear Editor,

The exosome, a kind of extracellular vesicle with a diameter in the range of 50–200 nm, has been extensively investigated in treating various skin issues, such as skin aging mitigation and cutaneous wound healing stimulation, and atopic dermatitis (AD).^1^, ^2^, ^3^ Compared to stem cell therapy, advantageous properties, such as a long half‐life, small size, favorable penetration, and low immunogenicity, make exosomes more favorable and beneficial.^4^ Although exosomes have been extensively investigated for their application in skin damage repair, the only sources shown to be effective are those derived from mesenchymal stem cells (MSCs).^5^ However, exosome isolation from MSCs accompanies multiple drawbacks, such as the time and effort required for MSC culture and expansion, the risk of contamination with red blood cells during the isolation process, neovascularization potential, diminished cell viability, and the extreme invasiveness of removing MSCs from bone marrow.^6^ Therefore, finding an alternative cell source for exosome isolation has become imperative for the effective application of exosomes in the repair of dysfunctional skin. Dermal fibroblasts (DFs) are the predominant cell type in the dermis and are responsible for regulating the extracellular matrix (ECM) while playing important roles in the maintenance of the normal structure and function of the skin. According to the previous study, DFs are considered a useful cell line in which to study exosome‐related treatments because these cells are easily cultivated. In this article, we suggest DFs for the extraction of exosomes because, in contrast with MSCs, fibroblasts more closely resemble the skin and may be obtained from the skin using less invasive techniques.^7^

This study thus aimed to determine if DF‐derived exosomes are a promising candidate for skin damage repair. To investigate the effects of DF exosomes on skin permeability barrier protection, we evaluated the expression levels of skin permeability barrier maintenance biomarkers in keratinocytes treated with 1‐chloro‐2,4‐dinitrobenzene (DNCB).

We harvested exosomes from conditioned media of fibroblast cells (Gibco, Grand Island, NY, USA) using differential ultracentrifugation. When the fibroblast cells reached ∼95% confluency in a 150‐mm petri dish, they were treated with cycloheximide (5–100 μg/ml) to generate a stress‐induced condition. Post‐treatment‐conditioned media were collected and subjected to differential centrifugation. Lastly, pellets containing harvested exosomes were separated and collected from the ultracentrifuged supernatant, resuspended in phosphate‐buffered saline, and stored at −80°C for further examination. Light microscopy revealed that the cultured fibroblasts exhibited a typical spindle/fibroblast‐like structure while adhering to the culture vessel surface (Figure 1A). The characterization of the harvested exosomes from the fibroblast cell culture‐conditioned media was done by nanoparticle tracking analysis (NTA). NTA was used to determine the particle number and size of the isolated exosomes. ZetaView (Analytik, Cambridge, UK) was used to determine the vesicle concentration and size distribution. The mean particle sizes of the isolated fibroblast exosomes were 215.4 ± 116.1 nm, with a concentration of 2.33×10^10^ ± 2.22×10^9^ particles/ml. (Figure 1B)

**FIGURE 1:**
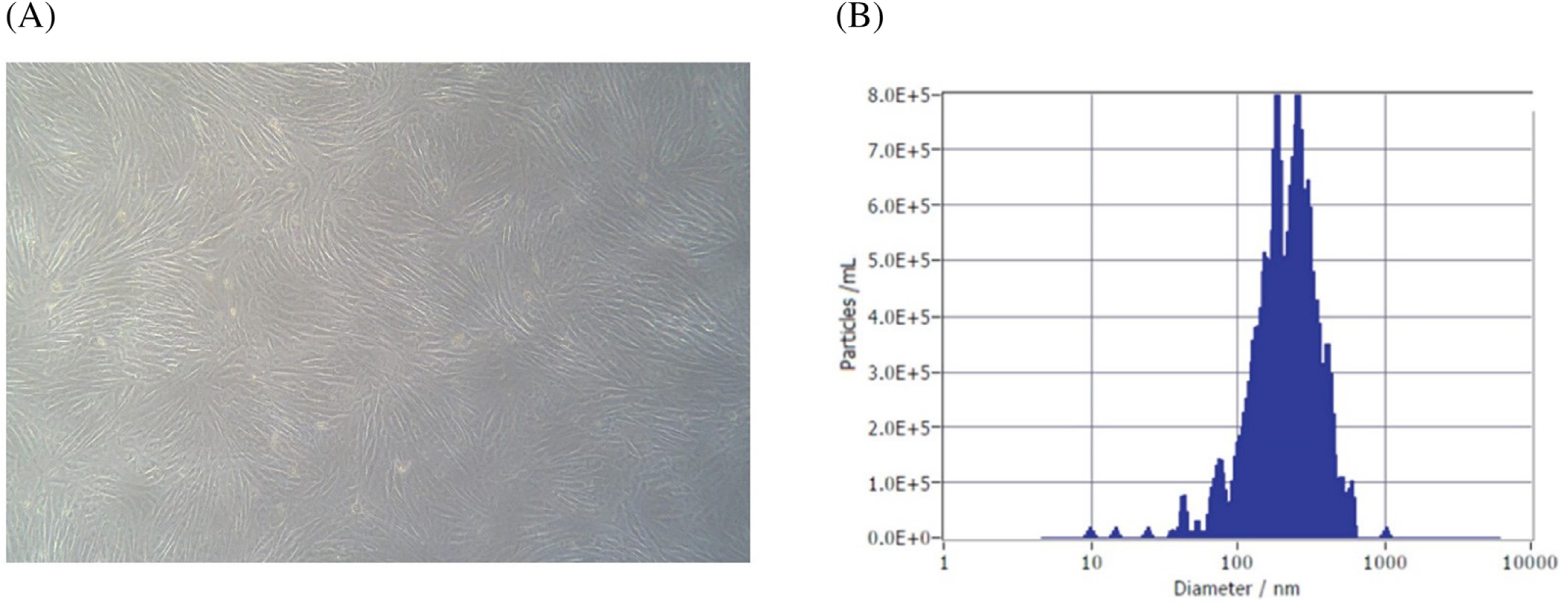
Characterization of dermal fibroblast cells and isolated exosomes. (A) The morphology of fibroblast cells observed under light microscopy. (B) The size distribution of isolated exosomes detected through nanoparticle tracking analysis.

The cytotoxicity of the isolated exosomes was least at concentrations of 1×10⁴, 1×10⁵, and 1×10⁶ particles/ml after 24 and 48 h of treatment. However, when the exosome concentration was 1×10⁷ particles/ml, we detected considerable cytotoxicity after 24 h of treatment. Interestingly, the cytotoxic effects on the HaCaT cells were further increased after 48 h of treatment with the harvested exosomes. (Figure 2)

**FIGURE 2:**
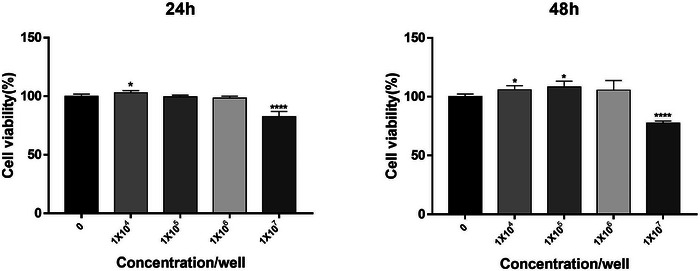
Human skin keratinocytes (HaCaT) were purchased from AddexBio (San Diego, CA, USA). HaCaT cells were cultured in DMEM, along with 10% fetal bovine serum (FBS) and 1% antibiotic‐antimycotic (AA) solution in a 37°C, 5% CO_2_ incubator. Every 48 to 72 h, fresh DMEM was used to replenish the cell culture media. The cytotoxicity of the fibroblast‐derived exosomes was assessed using 3‐(4,5‐dimethylthiazol‐2‐yl)−2,5‐diphenyltetrazolium bromide (MTT; Sigma‐Aldrich, St. Louis, MO, USA). HaCaT cells were distributed into 96‐well plates (1×10⁴ cells/well) and incubated for 24 h under cell culture conditions (37°C, 5% CO_2_). Then, the medium was replaced with a supplement‐free medium, and different exosome concentrations of 1×10^4^, 1×10⁵, 1×10⁶, and 1×10⁷ particles/ml were added to the cells and incubated for 24 and 48 h. Post‐exosome addition, the cells were treated with 5 mg/ml MTT solution (Sigma‐Aldrich, St. Louis, MO, USA; M5655) and incubated in a 37°C incubator for 4 h. After incubation, the supernatant was removed, and the formazan formed by MTT reduction was lysed by adding dimethyl sulfoxide (DMSO; MilliporeSigma, Billerica, MA, USA, 1.02952). The absorbance was then measured at 540 nm using a spectrophotometer (Molecular Devices, San Jose, CA, USA).

Exosome treatment increased the protein levels of skin permeability barrier biomarkers, compared to the control in HaCaT cells. However, the optimal concentration at which this epidermal barrier protein was expressed was 1×10⁴ particles/ml (Figure 3A). The expression level of the epidermal barrier proteins was significantly reduced in the DNCB‐treated HaCaT cells compared to the control. Intriguingly, exosome treatment at 1×10⁴ particles/ml increased the protein expression level of all skin permeability barrier biomarker proteins in comparison to other concentrations (Figure 3B). In the case of FLG, all three exosome concentrations (1×10⁴, 1×10⁵, and 1×10⁷ particles/ml) were shown to substantially recover the diminished protein expression level. However, in the case of IVL, LOR, and HAS1, only the 1×10^4^ particles/ml concentration effectively and greatly restored the diminished epidermal marker protein level compared to the remaining exosome concentrations. This data led us to conclude that 1×10^4^ particles/ml is the most optimum, safe, and effective exosome concentration to be used clinically for the repair of damaged skin cells.

**FIGURE 3:**
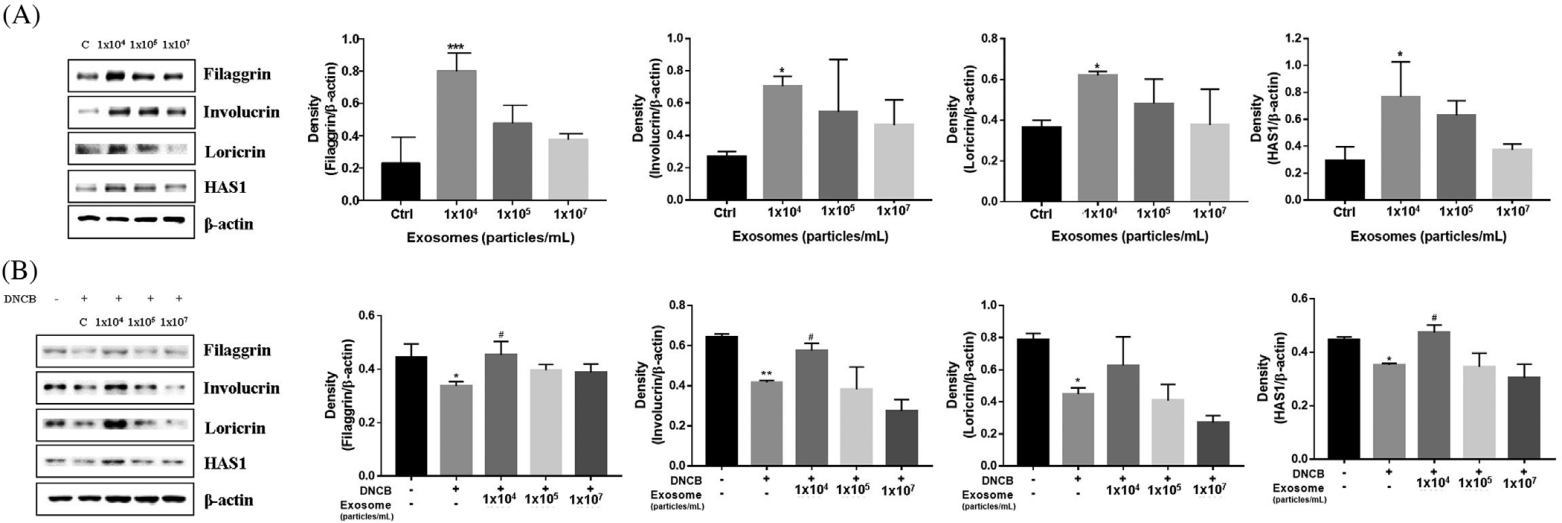
To evaluate the effect of dermal fibroblast (DF)‐derived exosomes on the expression of biomarkers associated with skin barrier functions, FLG, IVL, LOR, and HAS1 expression in human skin keratinocytes (HaCaT) cells was assessed through immunoblotting. (A) Protein expression analysis of human keratinocytes (HaCaT cells) treated with fibroblast‐derived exosomes. (B) Protein expression associated with fibroblast‐derived exosome treatment of 1‐chloro‐2,4‐dinitrobenzene (DNCB)‐treated human keratinocytes.

Recently, the effects of exosomes on various skin diseases have been extensively studied. The key benefits of exosomes are their high stability, non‐immune rejection, and direct stimulation of target cells. Especially, the miRNAs contained in the exosomes are engaged in the regulation of various cellular responses through binding to the 3′‐untranslated regions. Therefore, a single ingredient (exosomes) can contribute to multiple therapeutic effects. So far, multiple endogenous exosome types, such as those from adipose‐derived stem cells and mesenchymal stem cells, have crucial roles in aging, wounds, and AD.^7^, ^8^

Human DFs (HDFs) constitute the most predominant mesenchymal cell type in connective tissue, where collagen and elastic fibers of the ECM are deposited and are responsible for the maintenance of the normal structure and functions of the skin. As a result, very few studies have investigated the reparative effects of fibroblast‐derived exosomes, which stimulate the migration and proliferation of collagen and the synthesis of fibronectin and collagen I.^9^ In addition, exosomes derived from autologous DFs were found to more efficiently ameliorate senescence‐related tissue damage and promote cutaneous wound healing, which indicates that HDF‐exosomes can be potential candidates for protecting and repairing skin damage. Furthermore, exosomes isolated from conditioned media during three‐dimensional dermal fibroblast culture were shown to stimulate ECM synthesis and secretion from dermal fibroblasts, as well as cellular migration and proliferation, in part through interleukin‐6 signaling. These results imply that DF‐derived exosomes participate in numerous stages of dermal ECM formation, thus contributing to skin damage repair, and illustrating their potential mechanism.^10^ However, no study has investigated the mechanism used by DF exosomes on skin permeability barrier protection. This article attempts to evaluate the therapeutic significance of DF‐derived exosomes on skin epidermal marker proteins. Hence, we evaluated the expression levels of skin permeability barrier maintenance biomarkers in keratinocytes treated with DNCB, a skin irritant, and inducer of dermatitis, including AD.

Though exosome cytotoxicity has been the subject of some investigations, it is now believed that exosomes generated from fibroblasts do not exhibit considerable cytotoxicity. This is probably because exosomes are naturally secreted cell‐derived vesicles that are engaged in regular physiological functions.^4^ However, it is still essential to emphasize that the safety and efficiency of fibroblast‐derived exosomes in clinical settings require further investigation. In our study, the cytotoxicity of the harvested exosomes from DFs using the MTT assay was evaluated before determining their repair efficacy. The optimal concentration was identified from four different exosome concentrations (1×10^4^, 1×10⁵, 1×10⁶, and 1×10⁷ particles/ml). Our data showed that DF‐derived exosomes at concentrations of 1×10^4^, 1×10⁵, or 1×10⁶ particles/ml displayed the least cytotoxicity after 24 and 48 h of treatment (Figure 2).

Downregulating the expression of IVL, LOR, FLG, and HAS1 is the hallmark feature of AD skin lesions and is associated with epidermal barrier dysfunction.^8^ This study reveals the positive effects of DF‐derived exosomes on skin cells where exosome treatment of keratinocytes stimulates the expression of the epidermal marker proteins FLG, LOR, HAS1, and IVL. In this work, we established an AD model of keratinocytes treated with 5 μM DNCB to investigate the skin‐repairing effectiveness of fibroblast‐derived exosomes on DNCB‐treated skin. Our finding showed the fibroblast‐derived exosomes restored the DNCB‐induced reduction in FLG, LOR, IVL, and HAS1 expression levels (Figure 3).

Notably, in this study, the optimal exosome concentration was determined, which showed the maximum efficacy in expressing biomarkers related to the integrity and inflammation of the permeability barrier in HaCaT cells. All three exosome concentrations (1×10^4^, 1×10⁵, and 1×10⁷ particles/ml) have shown significant effects on protein expression, but the 1×10^4^ particles/ml concentration was depicted as the optimum. Similarly, regarding the AD model of keratinocytes, the 1×10^4^ particles/ml concentration has been shown to be optimum for repairing damaged skin, which may be attributed to several factors. A possible hypothesis is that the exosomes may become exceedingly concentrated at higher amounts and may not be adequately absorbed by the recipient cells, leading to decreased efficacy.

In conclusion, fibroblast‐derived exosomes at a concentration of 1×10^4^ particles/ml were the optimum to show efficaciousness in increasing skin epidermal barrier proteins, thus increasing the recovery rate of skin damage without causing cytotoxic effects in this study. Based on these findings, we conclude that only fibroblast‐derived exosomes at specific concentrations can be of clinical use for their prospective application in treating skin barrier dysfunction diseases like AD. To our knowledge, this was the first study of its kind to investigate how DF‐derived exosomes recover skin barrier functions. This study might thus serve as a first step toward the development of fibroblast‐derived, exosome‐based treatments for AD clinically. Additionally, this study only used in vitro experiments to illustrate the effects of DF‐derived exosomes; hence, we plan to conduct further experiments and animal studies to delineate the exact mechanisms of action underlying the efficacy of DF‐derived exosomes.

## CONFLICT OF INTEREST STATEMENT

The authors declare no conflict of interest.

## FUNDING INFORMATION

None.

## ETHICS STATEMENT

Not applicable.

## Data Availability

Contact the corresponding author for data availability.
